# Separable Learning Systems in the Macaque Brain and the Role of Orbitofrontal Cortex in Contingent Learning

**DOI:** 10.1016/j.neuron.2010.02.027

**Published:** 2010-03-25

**Authors:** Mark E. Walton, Timothy E.J. Behrens, Mark J. Buckley, Peter H. Rudebeck, Matthew F.S. Rushworth

**Affiliations:** 1 Department of Experimental Psychology, University of Oxford, Oxford OX1 3UD, UK

**Keywords:** SYSNEURO

## Abstract

Orbitofrontal cortex (OFC) is widely held to be critical for flexibility in decision-making when established choice values change. OFC's role in such decision making was investigated in macaques performing dynamically changing three-armed bandit tasks. After selective OFC lesions, animals were impaired at discovering the identity of the highest value stimulus following reversals. However, this was not caused either by diminished behavioral flexibility or by insensitivity to reinforcement changes, but instead by paradoxical increases in switching between all stimuli. This pattern of choice behavior could be explained by a causal role for OFC in appropriate contingent learning, the process by which causal responsibility for a particular reward is assigned to a particular choice. After OFC lesions, animals' choice behavior no longer reflected the history of precise conjoint relationships between particular choices and particular rewards. Nonetheless, OFC-lesioned animals could still approximate choice-outcome associations using a recency-weighted history of choices and rewards.

## Highlights

► OFC lesions can impair decision making in both changeable and fixed environments ► OFC is critical for assigning credit for a particular reward to a particular choice ► OFC lesions spare system to approximate learning based on choice and reward history ► Role in contingent learning underlies impairments in flexible decision making

## Introduction

Learning, tracking, and updating the predictive value associated with environmental stimuli is essential to advantageous decision making. One region consistently implicated in the guidance of such adaptive choice behavior is the orbitofrontal cortex (OFC). It has been suggested that OFC is crucial for processing negative outcomes (Fellows, 2007; Kringelbach and Rolls, 2004) or for the ability to inhibit previously chosen actions (Chudasama and Robbins, 2003; Clarke et al., 2008; Dias et al., 1997; Elliott et al., 2000; Jones and Mishkin, 1972). Deficits in flexible adjustments of decision-making that are witnessed after OFC lesions are therefore often assumed to result either from either an insensitivity to the absence of rewards or a perseveration of choice.

To date, the cardinal tests of flexible reward-guided decision making have been two-option deterministic reversal learning tasks. It has been frequently demonstrated that OFC lesions impair performance following such reversals, even though the initial stimulus-outcome discrimination learning appears unaffected (Butter, 1969; Clarke et al., 2008; Dias et al., 1997; Fellows and Farah, 2003; Iversen and Mishkin, 1970; Izquierdo et al., 2004; Jones and Mishkin, 1972; Rolls et al., 1994; Schoenbaum et al., 2002). Both single-neuron and BOLD responses in this region also show rapid changes to reflect new associations when stimulus-reinforcement contingencies change (O'Doherty et al., 2003; Stalnaker et al., 2006; Tremblay and Schultz, 2000; Walton et al., 2004). Nonetheless, the precise role that OFC plays in this type of flexible decision-making is not clear. This is in part because such reversal tasks are limiting as they can often be solved using a simple rule-based strategy and do not require animals to continuously track the value of several independent alternatives to decide what to do.

**Figure 1:**
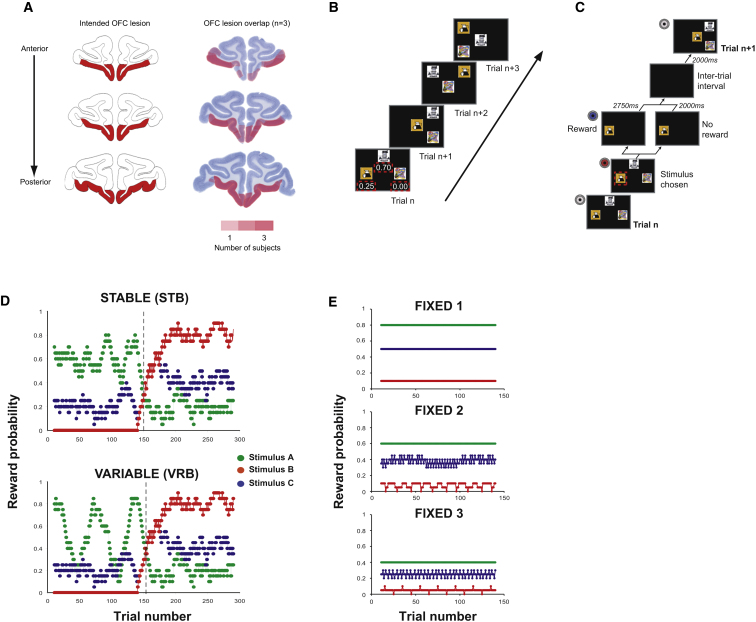
OFC Lesion Location and Task Schematic (A) Diagram of intended (left) and actual (right) OFC lesion locations. Redness of shading on the actual lesion diagram represents the number of animals (1–3) showing overlap at each location. (B and C) Schematic of trial-by-trial (B) and within-trial (C) task structure. On each trial, monkeys were presented with three clipart stimuli in one of four possible locations on a touchscreen (trials n to n+4). Each stimulus was associated with different outcome probabilities (example probabilities in red dashed boxes on trial n are shown for illustrative purposes only). On each trial, selecting one stimulus caused the other two options to extinguish and reward to be delivered according to the reward schedule. Gray, blue, and red circles = different 250 ms tones. (D and E) Predetermined reward schedules used in the changeable (D) and fixed (E) conditions. The schedules determined whether or not reward was delivered for selecting a stimulus (stimulus A–C) on a particular trial. Dashed black lines in (D) represent the reversal point in the schedule when the identity of the highest value stimulus changes.

Therefore, the present study was designed to reexamine the causal function of OFC in decision making in the context of changing reward values (Figure 1). Macaque monkeys performed different versions of a three-armed bandit task (Figures 1B–1E). In the first conditions, the reward associations of the three options could change both gradually and independently of one another meaning that fluctuations in outcome assignments occurred both with and without reversals in the identity of the most highly rewarding option (Figure 1D). To explore the role of OFC even in unchanging probabilistic environments, in the remaining three conditions, reward assignments remained stable although the average reward rate of the task environment was manipulated so that animals had to integrate across more trials in some conditions than others to discover the identity of the most highly rewarding option (Figure 1E).

Two key sets of findings were made. First, despite replicating the observation that selective OFC lesions impaired decision-making following reversal in the identity of the best-rewarded option, finer-grained analyses of trial-by-trial choice behavior demonstrated that this was not due to a failure to inhibit previously rewarding responses or insensitivity to negative outcomes as lesioned animals were also just as able as controls to respond to local changes in reward likelihood when the identity of the best stimulus remained the same.

The second set of findings demonstrate that the overall pattern of impairments can be explained by considering the OFC as critically concerned with specific contingent learning, the process by which the credit for an outcome becomes assigned to the appropriate previous choice. This process is particularly taxed at times such as when reward contingencies change, where multiple different stimuli might be chosen and different outcomes witnessed (Seo and Lee, 2008).

It has long been known that, even during normal behavior, a given outcome can reinforce not just the choice that led to its delivery but also other responses that were made close in time, both preceding and even following this outcome (Thorndike, 1933). This “spread-of-effect” was visible in our control animals, though it was dwarfed by the tendency to associate an outcome with its correct, causal choice (i.e., appropriate credit assignment). By contrast, monkeys with OFC lesions exhibited a specific deficit in the ability to associate an outcome with its correct choice and, in doing so, unmasked their tendency to instead relate outcomes with choices that occurred close in time. This meant that their choice behavior was now predominantly driven by the association between the recent history of outcomes and recent history of rewards.

Such a facility to learn using recent choice and reward histories would allow animals to make accurate approximations of specific stimulus-outcome associations during periods when the same choice is made repeatedly for a constant rate of reward, but learning would be severely compromised when the pattern of choices and outcomes is variable, such as following a reversal. We demonstrate finally a key implication of this idea: OFC lesions impair learning even in environments where contingencies never change (Figure 1E), so long as initial credit assignment is made difficult by placing animals in a context where the average reward rates are lower, guiding animals to have a mixed history of choices between the available options. We therefore argue that a crucial role of the OFC is in learning and updating predictive contingent relationships between particular choices and consequent outcomes and that a failure in this faculty can account for the pattern of impaired decision-making seen after lesions to parts of primate OFC.

## Results

### Changeable Three-Armed Bandit Schedules: Reversal Behavior

To probe the specific function of the OFC during flexible decision making, we tested six macaques—three controls and three animals given selective OFC lesions after presurgical testing (Figure 1A and see Supplemental Information available online)—on two types of continuously varying three-armed bandit tasks where animals had to choose their responses based on stimulus-reward probabilities (Figures 1B–1D). At the start of each testing session, animals were presented with three novel stimuli, meaning that they had no information other than the reinforcement delivered following a choice to guide their estimates of the expected values associated with that option. Whether or not reward was received for a particular stimulus choice was controlled by pre-determined outcome schedules. In the first set of experiments, two comparable outcome schedules were used—“Stable” (STB) and “Variable” (VRB)—in which the likelihoods of each alternative leading to reward varied continuously over the session, with identity of the most rewarding option reversing half-way through (Figure 1D, right of dashed line). Trial-by-trial reward probabilities were fixed according to these schedules and were identical for each animal and in each testing session using a particular schedule.

**Figure 2:**
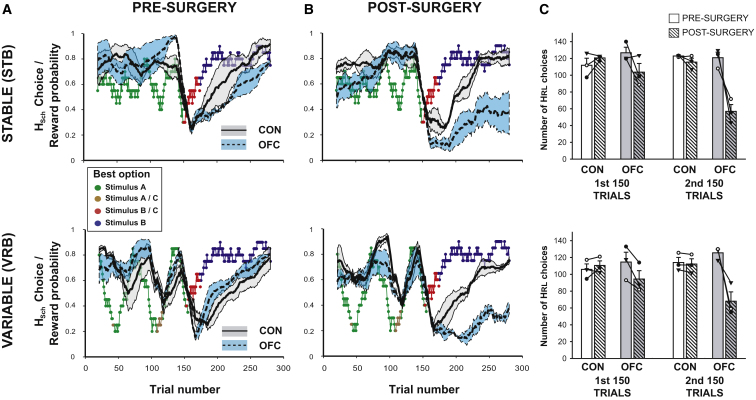
Likelihood of Choosing H_sch_ in STB (Upper Panels) and VRB (Lower Panels) (A and B) Average pre- (A) and postsurgery (B) choice behavior in the control (solid black line) and OFC groups (dashed black line). SEMs are filled gray and blue areas respectively for the two groups. Colored points represent the reward probability and identity of H_sch_ (stimulus A–C). (C) Average number of choices during the first or second 150 trials that were congruent with H_RL_ (the subjectively highest value option as defined by a reinforcement learning model). Controls, white bars; OFCs, gray bars. Symbols and connecting lines represent data for individual animals.

We analyzed the data based on both the “objective” value associated with each stimulus (based on a ±10 trial running average of the programmed reward probability, where the objectively highest value stimulus at any point in time was referred to as H_sch_) and on estimates each animal's “subjective” value (the experienced reward probabilities obtained using a simple Rescola-Wagner model with a Boltzmann action selection rule [Behrens et al., 2007; Sutton and Barto, 1998]), where the subjectively highest value stimulus at any point in time was referred to as H_RL_(see Experimental Procedures). Preoperatively, all animals rapidly learned to find the option with the highest probability of reward on both schedules (Figure 2A), and following the reversal of the identity of the H_sch_ option at around trial 150, animals then altered their pattern of choices to discover the new H_sch_. There was no difference in the rates of selection of the highest value option in the two groups defined either by H_sch_ or H_RL_ (all p > 0.14). Importantly, too, there was no effect of testing session (all p > 0.2), suggesting that animals did not develop a model of the underlying task structure.

Following surgery, there was a dramatic change in choice patterns, with the OFC-lesioned group failing to find and persist with the new H_sch_ option following reversal on both schedules (Figure 2B). When the data were divided up into the initial learning and tracking phase (first 150 trials) and the reversal phase (second 150 trials), there was a significant three-way Lesion Group × Surgery × Phase interaction in the H_RL_ data (F_1,4_ = 25.8, p = 0.007), which post hoc tests showed was driven by the fact that the OFC group was only significantly worse at choosing the H_RL_ option than the controls postoperatively during the reversal phase in both conditions (p = 0.001) but not during any other period of testing (this was also true for H_sch_: p = 0.008 for difference between the groups during the postoperative reversal phase, p > 0.2 otherwise; Figure 2C).

### Changeable Three-Armed Bandit Schedules: Initial Learning, Value Tracking, and Choice Alteration

The above analyses demonstrated that there was no statistically-evident alteration in choice behavior during the first 150 trials of either schedule when OFC-lesioned animals initially had to learn and track the highest value stimulus in either condition. A further analysis investigating the average number of trials to reach a criterion of >65% H_sch_ choices prior to the reversal also found no significant differences in the rate of learning pre- or postoperatively (Mann-Whitney test: p > 0.12 in both conditions). This is comparable to several previous lesion studies to have reported effects following reversals along with intact discrimination learning (Clarke et al., 2008; Izquierdo et al., 2004; Schoenbaum et al., 2002).

Especially notable is that choice performance of the OFC-lesioned group is comparable to that of the control animals even in the VRB schedule when the local likelihood of H_sch_ resulting in reward is fluctuating markedly. This rapid behavioral response to local changes in the rate of both negative and positive feedback would appear to contradict several accounts of OFC function during flexible decision-making that have suggested that this region is important for detecting negative feedback in order to subsequently adjust behavior (Fellows, 2007; Kringelbach and Rolls, 2004).

**Figure 3:**
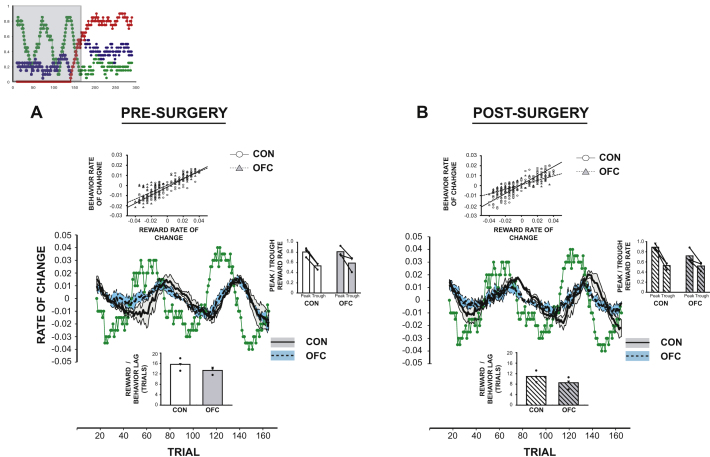
Tracking Value during the First 150 Trials of the Variable Schedule Responsiveness of choice behavior to changes in reward likelihood of the highest value stimulus during the first 150 trials of VRB schedule (shaded area in upper inset) both before (A) and after (B) surgery. Main figure depicts rate of change of reward likelihood (green points) along with rate of change of behavior in controls (solid black line; gray shading = SEM) and OFCs (dashed black line; blue shading = SEM). Inset graphs show the average peak and lowest rates of choosing the highest value stimulus (right panel), the lag between changes in reward likelihood and behavior (lower panel), and the relationship between the rate of change of reward likelihood and of delagged choice behavior (upper panel). Controls, white bars; OFCs, gray bars.

To explore this, we examined three measures of stimulus-outcome sensitivity and flexible performance during this first phase of VRB: (1) the lag in trials between the H_sch_ reward rate fluctuating and H_sch_ choice performance changing (lower inset panel in Figures 3A and 3B), (2) the relationship between change in H_sch_ choice performance and H_sch_ reward rate fluctuations (adjusted for the above average lag in performance; upper inset panel in Figures 3A and 3B), and (3) the difference in the average highest and lowest proportion of H_sch_ choices during fluctuations in H_sch_ likelihood (right-hand inset panel in Figures 3A and 3B). These analyses together probe the degree to which OFC-lesioned animals are able to respond to changes, particularly decrements, in the local reward rate.

There was no difference in how quickly the OFC-lesioned group on average responded to a local change in H_sch_ following surgery (measure a: Lesion Group × Surgery: F_1,4_ = 0.01, p = 0.98), and there was also no significant reduction in the sensitivity of the relationship between H_sch_ choice performance and H_sch_ reward rate postoperatively in the OFC group (as indexed by the slope relating these two parameters; measure b: Lesion Group × Surgery: F_1,4_ = 1.95, p = 0.26). Similarly, there was no significant reduction in the average range of H_sch_ choices in the two groups (measure c: Lesion Group × Surgery: F_1,4_ = 1.65, p = 0.27). Taken together, this demonstrates that during the first phase of VRB, the OFC-lesioned monkeys could track local changes in reward rates of the currently selected option, both when there was an increase in negative or positive feedback for selecting the best option, militating against any theory emphasizing the role of OFC in simply responding to negative feedback.

**Figure 4:**
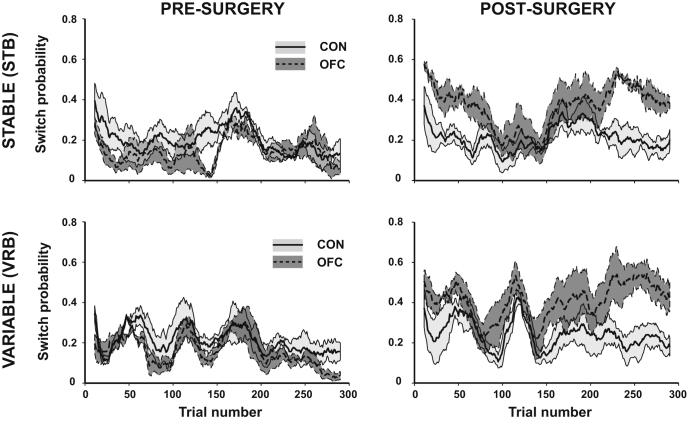
Rates of Switching Behavior during the Changeable Schedules Pre- and postsurgery average trial-by-trial switching likelihood across STB and VRB in control (solid black line, light gray shading = SEM) and OFC (dashed black line; dark gray shading = SEM) animals. See also Figure S1.

Such flexible behavior would also appear to rule out the notion that OFC lesions cause inflexible or perseverative responding in the face of changes in reinforcement (Elliott et al., 2000). This conclusion is bolstered by analyses of the trial-by-trial patterns of choice alternation behavior in the two groups. The point when OFC-lesioned animals were exhibiting impairments during the reversal phase on both schedules was actually associated with a local *increase* in the rate of switching between the alternatives (Figure 4). Overall, the lesioned monkeys were on average 1.6–4.7 times more likely to change their stimulus selection compared to the previous trial than prior to the lesion across the testing schedules (Lesion Group × Surgery: F_1,4_ = 8.66, p = 0.042; Figure 4). This was even the case examining just the 50 trials immediately postreversal in the two schedules (p = 0.032), underlining that any deficit here could not be caused by perseveration. Moreover, further analyses demonstrated that the OFC-lesioned animals' increased rate of switching was not a consequence of the reversal deficit causing these animals to receive less frequent rewards and was not modulated by receipt or absence of reward (Figure S1).

To summarize, the findings replicate previous studies demonstrating reversal deficits following a switch in the identity of the H_sch_ in OFC-lesioned animals accompanied by a largely intact ability to make appropriate choices when initially learning the values of the options. However, these same lesioned animals were able to track local changes in value of the currently chosen option. Rather than being caused by insensitivity to negative feedback or an inability to update response strategies, the OFC group's impairment was the result of an increased propensity to alternate between the different available options.

### Specific Contingent Learning in a Changeable, Multioption Environment

OFC lesions cause profound deficits in flexible alterations of behavior. While such flexible learning must indeed be reliant on reward processing, it also has a determinant that is perhaps even more fundamental: the understanding of the *causal* relationship between a particular choice and its contingent outcome.

It has long been known that choices closely followed by reward are more likely to be repeated on subsequent occasions whereas those followed by aversive consequences become likely to be avoided (“Law-of-Effect,” Thorndike, 1911). However, it is frequently overlooked that rewards do not just reinforce the choices that lead to them but also reinforce other choices made contiguously, either in the recent past or even those closely *following* on subsequent occasions. Such choices, even though they are just temporally contiguous with reward, rather than causally responsible for reward, are often repeated (“Spread-of-Effect,” Thorndike, 1933; see also White, 1989). In runs of repeated choices, it is possible that such a mechanism could drive learning even in the absence of any direct association between choice and outcome. However, such a mechanism would be particularly inflexible in situations where choices or reward contingencies changed over time as ambiguities would exist as to which stimulus had caused which outcome.

**Figure 5:**
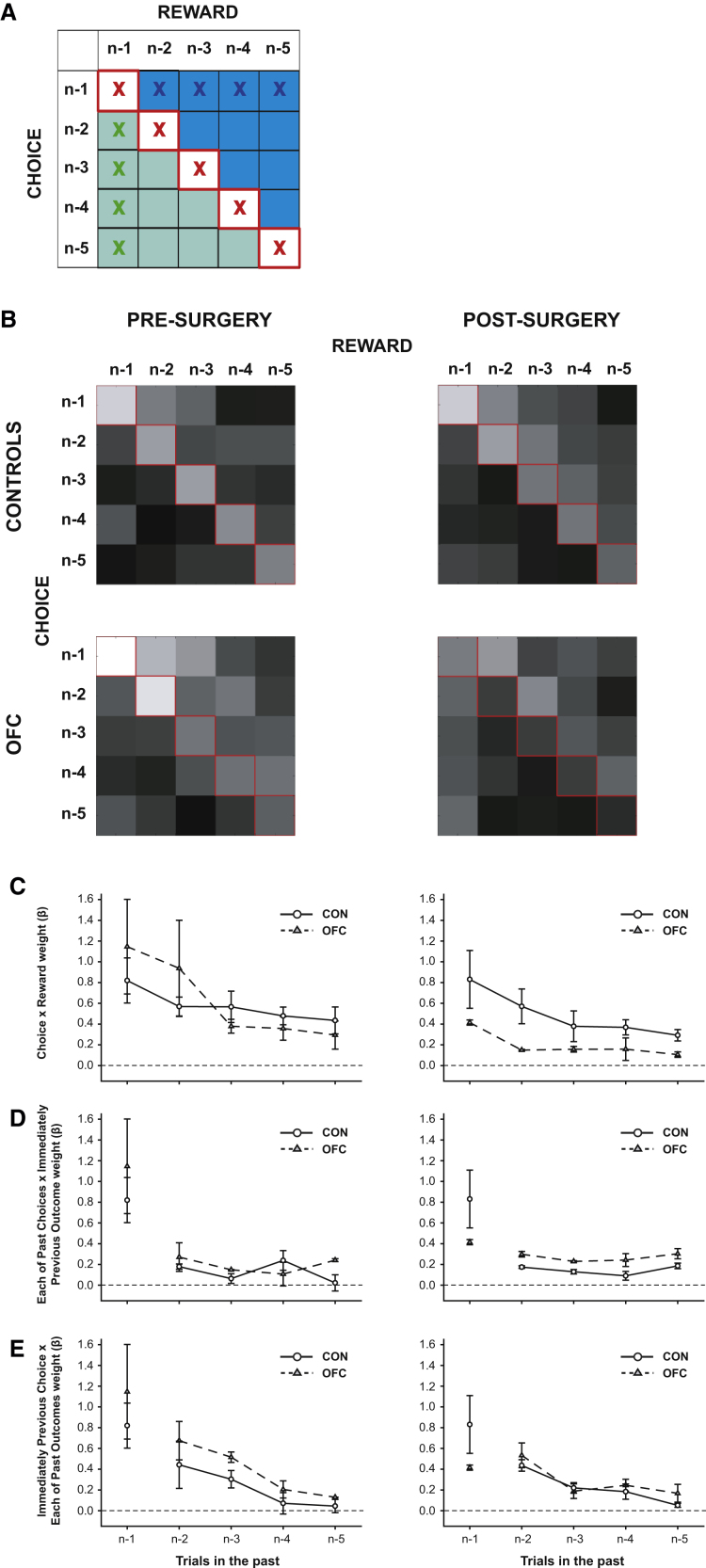
Influence of Recent Choices and Recent Outcomes on Current Behavior (A) Matrix of components included in logistic regression. Red (i), green (ii), and blue (iii) X's respectively mark elements representing the influence of: (i) recent choices and their specific outcomes; (ii) the previous choice and each recent past outcome, and (iii) the previous outcome and each recent past choice, on current behavior. Green area represents influence of associations between choices and rewards received in the past; blue area represents the influence of associations between past rewards and choices made in the subsequent trials. (B) Regression weights for this matrix for each group pre- and postoperatively, log-transformed for ease of visualization (bright pixels = larger regression weights). (C–E) Plots of influence of X-marked components in (A). The data for the first trial in the past in (C)–(E) are identical. Symbols and bars show mean and SEM values for controls (black circles, solid black lines) and OFCs (gray triangles, dashed gray lines). See also Figures S2 and S3.

It is therefore possible that the characteristic reversal deficit associated with OFC lesions is caused by an inability to associate a particular choice with a particular outcome. Both lesion and single-unit studies have suggested that OFC might carry a representation of the choice that was made when outcomes are received (Meunier et al., 1997; Tsujimoto et al., 2009). In order to test this idea, we ran a multiple logistic regression analysis (Barraclough et al., 2004; Lau and Glimcher, 2005) to see which combination of factors best explained animal choices. We included as regressors in the analysis all of the possible combinations of choice and outcome in the recent past (trials n-1 to n-5), along with a confound regressor for trial n-6 to capture longer term choice/reward trends (Supplemental Information; Figure 5A). This allowed us to investigate the influence of specific choice-outcome associations on current behavior (red crosses, Figure 5A). Importantly, it was also possible to extract information about potential false associations as the value of an outcome is assigned backward based on choices made in previous trials (green area, Figure 5A) and as the value of an outcome spreads forward to choices made in subsequent trials (blue area, Figure 5A). In order to have adequate data to get accurate estimates of the strength of influence of these factors, we included data from both STB and VRB and two other analogous three-armed bandit schedules (Rudebeck et al., 2008; Figure S2).

Preoperatively, all animals' choices were strongly influenced by the stimuli they had recently selected and by the outcomes received for each of those choices, an effect that diminished with increasing separation from the current trial (Figures 5B, 5C, and S3). Prior to surgery, therefore, animals were able to associate specific choices with resulting outcomes. However, there was also a smaller influence of both the interaction between the previous reward and choice history (Figure 5D) and, for a few trials into the past, between the previous choice and reward history (Figure 5E). Hence, preoperative animals exhibited “Spread-of-Effect,” being likely to associate outcomes with unrelated choices made near in time.

Following surgery, the influence of specific choice-outcome associations on behavior was profoundly reduced postoperatively, an effect that was particularly prominent on trials near to the current one (Lesion Group × Surgery × Past Trial: F_4,16_ = 3.37, p = 0.035; Figures 5B and 5C). OFC-lesioned animals therefore demonstrated a significant impairment in the ability to use the direct association between a specific choice and its resultant outcome to guide choice behavior. Unlike these specific associations, OFC lesions caused no effect on the degree to which animals associated the previous outcome with the choices made in the past (Figure 5D; interactions including Lesion Group × Surgery: F < 2.37, p > 0.12) or the degree to which they associated the past rewards with the previous choice (Figure 5E; interactions including Lesion Group × Surgery: F < 0.93, p > 0.62). Individual analyses of the postoperative data showed that there was a significant influence on current choices in both groups of associations between both the latest outcome and choice history and between the previous choice and reward history (Controls: both F_1,2_ > 263.99, p < 0.005; OFCs: both F_1,2_ > 20.03, p < 0.047).

This logistic regression analysis implies that OFC-lesioned animals are able to process the outcomes of choices but show a profound impairment in associating these outcomes with the relevant preceding choice on which they were contingent, instead forming an association between their overall integrated history of choices and an overall integrated history of outcomes. This theory makes explicit predictions of situations in which OFC lesioned animals should exhibit counterintuitive and counterproductive behavior. In the following sections, we examine these situations in detail. In brief, the theory predicts that OFC animals will perform like controls in situations where the integrated recent history of choices is strongly predictive of each individual choice, and the integrated history of rewards is strongly predictive of each individual reward.

If OFC-lesioned animals are using their history of choices and outcomes, rather than particular conjoint choice-reward associations, to update their value estimates for each option, this group should also then exhibit a particular pattern of deficits when a new stimulus is chosen (for example, option B) after long history of choice on another stimulus (i.e., option A), as is the case in reversal learning. (Note that options “A,” “B,” and “C” do not necessarily directly refer to stimuli A, B and C, as depicted in Figure 1, but instead to sequences of similar choices). To investigate this hypothesis, we examined the effect of an outcome—reward or no reward—on a newly chosen stimulus, after various different histories of choices. If credit is correctly assigned, animals should always be more likely to reselect B on the following trial (n) if its choice on the previous trial (n-1) was rewarded than if it did not result in reward. By corollary, they should be less likely to switch back to A after B's that are rewarded than those that are not. Moreover, this effect should of course be *independent* of choice history if all credit is properly assigned to the new choice, B. By contrast, if the credit for the new outcome is assigned not to the choice that causes the outcome, but instead to the integrated history of choices, then reinforcement for a reward for choosing option B will be assigned partly based on previous choices of option A. Importantly, as the length of previous choice history on A increases, a reward on B should make the animals *less likely to choose B and more likely to choose A*, as the credit for this outcome is falsely attributed to A.

**Figure 6:**
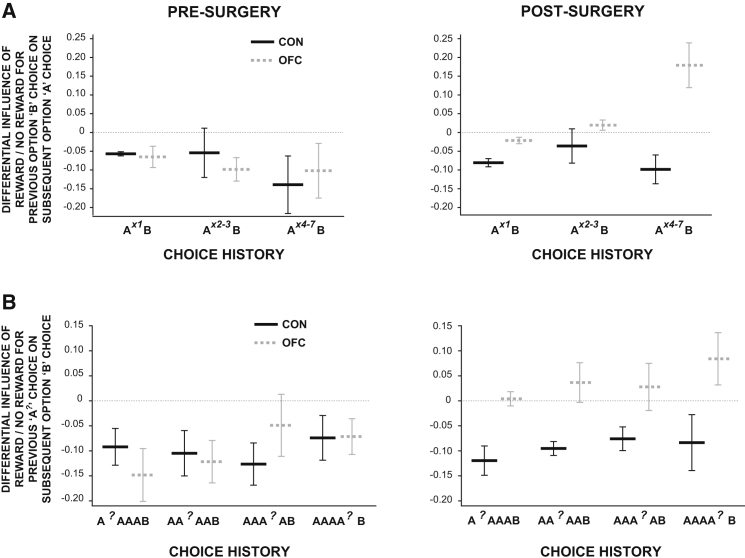
Influence of Past Choices (A) and Rewards (B) on Current Choice in Changeable Three-Armed Bandit Tasks (A) Difference in likelihood of choosing option A on trial n after previously selecting option B on trial n-1 as a function of whether or not reward was received for this choice. Data is plotted based on the length of choice history on A (1 previous choice of A, left plots; 2–3 previous choices of A, middle plots; 4–7 previous choices of A, right plots). See also Figure S4. (B) Difference in likelihood of choosing option B on trial n after previously selecting option A on trials n-2 to n-5 and option B on the previous trial (n-1), as a function of whether a particular previous A choice (A*^?^*) was or was not rewarded. Bars show mean and SEM values for controls (solid black lines) and OFCs (dashed gray lines). See also Figure S5.

Figure 6A (and Figure S4) shows just such an effect. We plotted the difference between trials following a reinforced B and those following an unreinforced B (trial n-1) in the likelihood that an animal will switch back to A (trial n). (Note that the likelihood of a reward on B being preceded by a rewarded A choice is not affected by the length of the previous choice history and is no different between the groups [both p > 0.27].) As predicted, preoperatively the effect of reward on B is to make them less likely to switch back to A (Figure 6A) and more likely to reselect B (Figure S4). However, the OFC-lesioned animals exhibited a very different pattern of responses (Lesion Group × Surgery × Trial^n-1^ Reward: F_1,4_ = 7.69, p = 0.050), with these animals post-surgery showing both a significantly increased propensity to switch back to option A after a reward on B and a decreased tendency to switch back to A after *not* receiving a reward on B (post hoc tests, both p < 0.05; Figure S4). Analyzing just the postoperative data, there was also a three-way interaction between Lesion Group × Trial^n-1^ Reward × Choice History (F_2,8_ = 5.68, p = 0.029), caused by the fact that this effect became more prominent the longer the previous choice history on A.

Such behavior—a tendency to link the reinforcement received on the previous trial to the recent history of choices—was not simply caused by an increase in random choices in the OFC-lesioned animals. After any history of A choices, a reward for choosing option B made a subsequent C selection *less likely in both groups* (main effect of Trial^n-1^ Reward: F_1,4_ = 18.79, p = 0.012; no interactions between Lesion Group × Surgery, all F < 1.1, p > 0.37; Figure S4). Taken together, this demonstrates that the OFC-lesioned animals are updating stimulus-value representations based not on the specific association between a choice and its outcome but instead partially based on the history of recent choices and an outcome.

Similar predictions can be made if the OFC-lesioned animals are acting on an integrated history of *rewards* as opposed to updating associations on the basis of the most recent reward. For example, reward delivered at trial n-2 in the lesioned animals should be associated not only with the choice made at n-2, but also with the subsequent choice made at trial n-1(compare, in Figures 5B and 5E, the influence of an association between the outcome on trial n-2 with the choice on trial n-1 with specific choice-outcome associations). This leads to the counterintuitive prediction that a new “B” choice should be more likely to be repeated if a previous “A” choice were rewarded than if not. Precisely this effect can be observed in Figures 6B and S5. We extracted patterns of choices where animal selected the same option (e.g., A) on at least 4 occasions (trial n-2 to ≥ n-5) and then changed to a new option (e.g., B; at trial n-1). We then examined the probability that the animal would reselect the new option (B; at trial n) as a function of reward being delivered on one of the previous A choices.

Control animals were always more likely to select A, and less likely to select B or C, at trial n if a previous A choice had sometime been rewarded than if not, regardless on which previous trial a reward was delivered on A. The credit for a reward delivered after an A choice was predominantly being correctly assigned to stimulus A. Moreover, this effect was cumulative such that their likelihood of returning to an A choice at trial n increased as more of the previous A trials (n-2 to n-5) were rewarded. By contrast, the OFC-group showed a markedly different effect postsurgery such that, compared to when no reward was delivered for a recent A choice, a recent reward on A *decreased* the likelihood of choosing A and significantly *increased* the likelihood of persisting with option B than before the lesion (Lesion Group × Surgery × Option A Reinforcement: F_1,4_ = 18.64, p = 0.012). The reinforcement after the A choice was strongly affecting the value of a subsequent B choice despite the fact that the reinforcement occurred *before* the B choice (Figure 6B). Similarly, these animals' choices on trial n of options A or B (though, importantly, not of C) were significantly less influenced by the frequency of rewards for previous A choices than controls (e.g., switch to A: Lesion Group × Surgery × Past Option A Reinforcement Frequency: F_2,8_ = 6.43, p = 0.022).

When this effect was broken down, we found that it was mainly driven by a decrease in the OFC-lesioned animals' likelihood of choosing option B again following a recent choice of A that was not reinforced (post hoc test: p = 0.003), although there was also a trend in this group for an increase in the likelihood of persisting with B following a recent reward for A (post hoc test: p = 0.061; Figure S5). Inspection of Figure 6B shows that the effect was particularly prominent the closer in time the reward on A occurs to the option B choice. Nonetheless, there was no significant interaction between Lesion Group and the number of trials elapsed since the Past Reward Trial as inspection of Figure 6B shows that there is a similar, if less marked effect also in the controls.

Comparable examples of where the OFC-lesioned animals' choices either resembled or predictably diverged from control groups' choices as a function of recent reward, and choice history could also be observed when examining alternation behavior. OFC-lesioned animals were significantly more likely to switch than controls after only 1–2 choices of the same option (p < 0.05), but rates of persistence became indistinguishable following 3 or more choices of one stimulus (Supplemental Results; Figure S6).

Taken together, the evidence suggests that the choices OFC-lesioned animals make are strongly influenced by their recency- and frequency-weighted history of past choices and of previous rewards. Although OFC-lesioned animals appear unable to make and update specific associations between the stimulus they chose and the outcome they received, they are just as able to use the contiguity between recent choices and rewards to select what responses to make. Such stimulus-outcome approximations would result in the animals learning accurate value representations when either their reward and/or choice histories are relatively constant, such as during the first 150 trials of STB and VRB, but will lead to aberrant learning when reward and choice histories are mixed and changeable, as exemplified by reversal situations where the values of stimuli alter such that the identity of the highest value stimulus changes.

### Fixed Three-Armed Bandit Schedules

**Figure 7:**
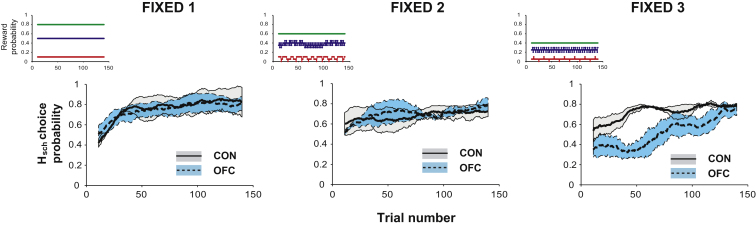
Likelihood of Choosing H_sch_ in the Fixed Three-Armed Bandit Schedules Controls, solid black lines (gray shading = SEM); OFCs, dashed gray lines (blue shading = SEM). Inset panels depict each predetermined reward schedule.

While the emphasis in many theories of OFC function has been on guiding choices in changeable environments, the evidence presented here indicates that this may be the result of a critical role for this structure in guiding specific contingent learning between stimulus-based choices and their outcomes which is severely taxed during reversal learning. Contrary to previous examples of intact discrimination learning in OFC-lesioned animals, it should therefore be possible to observe deficits following OFC lesions even using *fixed* schedules of reinforcement where there are never any reversals if it is made more difficult to determine the best option by lowering the reward likelihood of H_sch_, therefore requiring animals to integrate across more choices to determine which option was best. To investigate this, we tested the control and OFC-lesioned monkeys on three new fixed three-armed bandit schedules (Figure 1E). The ratio of likelihoods of the three options was the same in each condition, but the overall yield differed, with the options in FIXED 2 or FIXED 3 schedules rewarding at 0.75 and 0.5 times the rate as in FIXED 1 (Figure 7).

Using comparable analyses to those employed with STB and VRB, we investigated the speed of learning to choose H_sch_ on ≥65% of trials. While OFC-lesioned animals determined the identity of H_sch_ in a similar number of trials in FIXED 1 and 2 (Mann-Whitney test: both p > 0.8), they were significantly impaired at finding this in FIXED 3 which had the lowest rate of reinforcement, even though the values associated with the stimuli were constant throughout (Mann-Whitney test: p = 0.05). However, although slower at learning, the OFC group did eventually reach a comparable level of performance as controls. When the session was divided up into 2 halves (first and second 75 trials) the OFC group made fewer H_sch_ responses in FIXED 3 during the first half of the session, when learning the identity of the best stimulus (main effect of group: F_1,4_ = 7.97, p = 0.048), but they were no different from the control group during the second half (main effect of group: F_1,4_ = 1.64, p = 0.27). Also as in the changeable schedules, this deficit manifested itself in increased patterns of choice alteration that were particularly evident in FIXED 3 (Condition × Group: F_2,8_ = 5.64, p = 0.030, post hoc tests showed significant difference between switching in FIXED 3 and the other schedules).

## Discussion

OFC has long been associated with enabling animals to alter their behavior in response to changes in reinforcement, particularly during reversals in stimulus-outcome associations (Fellows, 2007; Murray et al., 2007; Schoenbaum et al., 2007). The current study replicated this finding using a changeable, stochastic three-armed bandit paradigm, with OFC-lesioned animals being markedly slower to update their choices than controls when the identity of the highest value option reversed (Figure 2). However, in contrast to several accounts (Dias et al., 1997; Fellows, 2007; Kringelbach and Rolls, 2004), this deficit was neither caused by insensitivity to positive or negative feedback and accompanying changes in reward rate (Figures 3 and S1), nor was it due to perseverative response selection (Figure 4).

If OFC lesions do not cause difficulties in reward monitoring or inhibiting previous responses, the obvious question arises as to what specific role the OFC plays in guiding adaptive decision making in a changeable environment. Our findings imply that its function is to guide contingent learning, a mechanism that allows rewards received for a particular choice among several alternatives to be correctly credited to that option alone (Figures 5 and 6).

Such an impairment in appropriate contingent learning may seem to be immediately contradicted by the fact that the large majority of previous studies have implicated OFC as only important following reversals in outcome associations but not during initial discrimination learning (Clarke et al., 2008; Fellows and Farah, 2003; Izquierdo et al., 2004; Schoenbaum et al., 2002) and that the OFC-lesioned animals' choice behavior in the current study was also largely unimpaired prior to the reversal. However, a second conclusion from our data is that OFC-lesioned animals are still able to rely upon alternative learning mechanisms which use representations of choice and reward history to approximate a link between the stimuli selected and outcomes received. This reliance results in the reinforcing effects of reward being assigned both backward based on the recency- and frequency-weighted history of choices (Figures 5A and S4) and also forward to choices made after an outcome is received (Figures 5B and S5).

A method of estimating stimulus value based on recent choice and reward histories, if fed into a response selection algorithm, would result in largely appropriate decision making in any situation when reward and choice histories are uniform; in other words when much of the recent choice history comprises of only taking one option and much of the recent reward history comprises of only one type of outcome (either reward or nonreward; Figure S1). Such uniform histories will be more likely when few alternatives are available or when the expected values of the options are far apart. It is just such conditions that prevail in the majority of studies of OFC in adaptive decision-making, the first half of the changeable three-armed bandit schedules (Figure 2), and in two of the three Fixed schedules tested here (Figure 7). However, when these conditions are not met, such as at a time of reversal (Figure 2) or, in the Fixed three-armed bandit schedule, where the absolute values were the lowest (Figure 7) then accurate learning is compromised. At such times, this method of approximating stimulus-reward associations will also often cause the values of the options to be closer together, leading to increased patterns of switching which, in turn, exacerbates the impairment (Figures 4 and S1).

Several lines of evidence support the idea that OFC impairment reflects the disruption of a mechanism for forming precise associations between particular choices and their resultant rewards, in the presence of an intact and simpler learning mechanism only capable of estimating such associations. First, data from logistic regression analyses demonstrated that the influence of precise paired associations between stimulus choices and reward outcomes, which was a strong predictor of choice behavior in control animals, was significantly reduced following OFC lesions (Figures 5B and 5C). By contrast, two other determinants of behavior remained, now unchecked, to influence decisions: first, recency-weighted associations between a received outcome and choices made before that outcome (Figure 5D), and second, recency-weighted associations between a chosen stimulus and rewards received before that choice (Figure 5E). While the overall weight of these factors was weaker in control animals than that of recent specific stimulus-outcome associations, both of these factors contributed to choices to an almost equal extent pre- and postsurgery in both the control and OFC groups. Therefore, it appears as if the approximation of stimulus-outcome associative learning used by the OFC group postoperatively is not a novel learning strategy to compensate for their deficit but instead is present in all animals. However, in normal animals, this “Spread-of-Effect” (Thorndike, 1933; White, 1989) will typically be dwarfed by the influences of knowledge of specific choice-outcome contingencies.

If it is the case that, without the guidance of specific stimulus-outcome associations, choice behavior is increasingly determined by the unmasked influence of choice history and recent reward, then this should have implications for the types of choices that are made. In controls and, prior to surgery, in the OFC group, a reward always increased the likelihood that animals would reselect the reinforced choice at the expense of others on later trials. However, following surgery in the OFC group, credit for a reinforcer was assigned not only to the choice that caused the reinforcement, but also to choices that had been made in surrounding trials (Figure 6). This effect was so pronounced that, after a long history of one choice (for example, option A), a new choice (i.e., option B) was *less likely to be reselected* if rewarded than if not (Figures 6A and S4); but also *more likely to be reselected* if the preceding A was rewarded than if not (Figures 6B and S5). In both cases, therefore, the credit for the reward was predominantly assigned to the wrong choice. However, this was done in a predictable fashion such that, rather than being assigned randomly, reinforcement was specifically distributed between choices that preceded and followed the reward.

Crucially, as in the controls, no credit was assigned to the third option (in this example, option C) that was absent in the local choice pattern. Reward for choosing either A or B made a future selection of C less likely in all circumstances (Figures S4 and S5). The specificity of the effect in relation to the recent choice history rules out any explanation based on undifferentiated increases in stimulus similarity or generalization after the lesions.

The claim that the OFC is essential for representing the conjunction of a particular reward with a particular choice having been made is consistent with several strands of evidence from previous studies. First, while there is an extensive literature demonstrating that OFC activity reflects information about anticipated and received reward value (Gottfried et al., 2003; Hare et al., 2008; Hikosaka and Watanabe, 2000; Schoenbaum et al., 2003; Tremblay and Schultz, 2000; van Duuren et al., 2008; Wallis and Miller, 2003) there are also data to show that OFC neurons dynamically encode information both about preceding and upcoming rewards (Simmons and Richmond, 2008). Second, as well as encoding outcomes, OFC maintains representations of currently relevant stimuli and choices over time or reactivates them at the time that reward is received (Lara et al., 2009; Meunier et al., 1997; Tsujimoto et al., 2009; Wallis and Miller, 2003). An extended role in contingent learning might also underlie an OFC contribution to maintaining expectations of specific outcomes and of future rewards across a delay. OFC lesions impair the ability of animals to alter their behavior toward a cue predicting a particular outcome if it has been devalued either by prefeeding or previously pairing it with nausea (Izquierdo et al., 2004; Ostlund and Balleine, 2007). Such a result might be expected if there were a deficit in representing the contingent link between different stimuli and their conjoint outcomes. OFC-lesioned animals fail to update associations correctly in situations when the outcome associated with one of two distinct stimulus-outcome pairings starts to be delivered non-contingently (Ostlund and Balleine, 2007). Similarly, changes in delay-based decision making (Rudebeck et al., 2006) may also result from a failure to generate at the choice point a representation of a future large reward contingent upon tolerating a delay as well as from incorrectly updating value representations when the contingent choice and outcome are separated in time or by other choices (as may be the case in the “credit assignment problem”).

OFC is well placed anatomically to mediate specific stimulus-reward learning. OFC, particularly lateral OFC which was the focus of the lesion in the current study (Figure 1A), is the recipient of afferents from high-level sensory areas in temporal and perirhinal cortex as well as of reinforcement information from limbic structures such as the amygdala (Carmichael and Price, 1995a, 1995b; Croxson et al., 2005; Morecraft et al., 1992). It is also one site of termination of dopamine fibers (Lewis, 1992) which could provide another source of information about expected value and deviations from such expectations (Schultz, 2007). OFC projects back to parts of the temporal lobe and amygdala, thus potentially allowing it to influence associative learning processes in these regions (Liu et al., 2000; Saddoris et al., 2005; Yanike et al., 2009). It may be important to understand the role that the OFC plays in contingent learning in the context of its relative specialization, in both the rodent and primate, for learning relationships between stimuli and outcomes rather than between responses and outcomes (Ostlund and Balleine, 2007; Rudebeck et al., 2008).

The conclusion that OFC is crucial for an aspect of stimulus-outcome learning and that this drives its role in reversal learning is consistent with the emphasis placed on OFC in associative learning by other researchers (Schoenbaum et al., 2007; Takahashi et al., 2009). The present study, however, emphasizes that more than one mechanism might mediate the association of stimuli with choices: an OFC-centered system for learning specific contingent stimulus-outcome pairings and at least one other more temporally-imprecise mechanism based on recent choices and outcomes that is spared in the OFC-lesioned animals. The notion that OFC-mediated reversal deficits are partly caused by the way remaining learning systems consequently function was previously implied by a study by Stalnaker and colleagues (2007) in which an OFC impairment was ameliorated following lesions to the amygdala, a structure known to be involved in aspects of associative learning.

While we and others have emphasized that there are many similarities between the structure and function of rodent and primate OFC (Price, 2007; Rushworth et al., 2007; Schoenbaum et al., 2006), it is nonetheless important to consider that cytoarchitectural studies indicate that primate OFC has expanded to include areas of granular and dysgranular cortex (Price, 2007; Wise, 2008), such as the relatively lateral OFC areas 11 and 13 that were the focus of the lesions in the current study. It is likely that other OFC regions, including more lateral, ventromedial or posterior agranular areas, may play subtly different roles in guiding stimulus-based learning and decision making (Butter, 1969; Fellows and Farah, 2003; Iversen and Mishkin, 1970).

In order for OFC-lesioned animals to be able to approximate associative learning based on recent choices and rewards, it is necessary that these elements should be represented in structures other than the OFC. Such signals have in fact proved to be relatively widespread and present in several brain areas, such as anterior cingulate cortex and striatum (Lau and Glimcher, 2007; Luk and Wallis, 2009; Seo and Lee, 2007, 2009). However, it is notable that these areas do not necessarily contain information about the conjoint history of rewards received in the context of particular choices, which may instead be a function of dorsomedial and lateral prefrontal cortex and lateral intraparietal cortex (Seo et al., 2007, 2009; Seo and Lee, 2009; Uchida et al., 2007). There is already a wealth of evidence for a multiplicity of learning systems in the brain (Balleine and Dickinson, 1998; Rangel et al., 2008; Weiskrantz, 1990). Our data provide evidence for a distinction between an OFC-based system for learning specific stimulus-reward contingencies and at least one additional extra-OFC system for reinforcement-based learning that incorporates recent choice history and the temporal contiguity of reward with subsequent choices.

In most studies to date, the contingencies between choices and reward have been straightforward, with limited available options and possible outcomes and with the associations between the two remaining stable. However, in many situations outside the laboratory, when there are frequently multiple alternatives and also delays between the consequences of responses and their causal antecedents, it is not straightforward to form appropriate associations. Learning in such complex situations can follow two distinct strategies: either through monitoring integrated choice and reward histories (for example, using eligibility traces: Sutton and Barto, 1998), or, where possible, through keeping track of individual choices and inferring precise associations between particular choices and rewards (Bogacz et al., 2007; Seo and Lee, 2008). This latter process likely requires an explicit encoding of the pertinent cues that have been encountered and the choices that have been made in order for reinforcement to update the appropriate predictors of eventual success or failure (Fu and Anderson, 2008). In the case of stimulus-reward learning, our findings suggest that the OFC may be crucial for the latter, but not the former, of these two strategies.

## Experimental Procedures

### Animals

Six adult male rhesus macaque monkeys (*Macaca mulatta*), aged between 4 and 10 years and weighing between 7 and 13 kg were used in these experiments. Three animals acted as unoperated controls, whereas the other three received bilateral aspiration OFC lesions following training and presurgical testing. All animals were maintained on a 12 hr light/dark cycle and had 24 hr ad libitum access to water, apart from when testing. All experiments were conducted in accordance with the United Kingdom Animals Scientific Procedures Act (1986).

### Behavioral Testing and Analysis

Prior to the start of experiments reported here, all monkeys had previous experience of using touchscreens and of other three-armed bandit tasks (see Rudebeck et al., 2008), though they had never performed with these particular schedules. On each testing session, animals were presented with three novel stimuli which they had never previously encountered, assigned to one of the three options (A–C). Stimuli could be presented in one of four spatial configurations and each stimulus could occupy any of the three positions specified by the configuration (Figure 1B). Configuration and stimulus position was determined randomly on each trial meaning that animals were required to use stimulus identity rather than action- or spatially based values to guide their choices. Stimulus presentation, experimental contingencies, and reward delivery was controlled by custom-written software (Figure 1C).

Reward was delivered stochastically on each option according to five predefined schedules: STB and VRB (changeable schedules) or FIXED 1–3 (fixed schedules; Figures 1D and 1E). The likelihood of reward for any option and of H_sch_ and H_RL_ choices was calculated using a moving 20 trial window (±10 trials). Whether or not reward was delivered for selecting one option was entirely independent of the other two alternatives. Available rewards on unchosen alternatives were not held over for subsequent trials. Each animal completed five sessions under each schedule, tested on different days with novel stimuli each time. For STB and VRB, the sessions were interleaved (i.e., day 1, STB1; day 2, VRB1; day 3, STB2; day4, VRB2; etc.) and data were collected both pre- and postoperatively. For the FIXED conditions, schedules were run as consecutive sessions, starting with the five sessions of FIXED 1, then five sessions of FIXED 2, and finally five of FIXED 3 and only postoperative data were collected. The changeable schedules comprised of 300 trials per session and the fixed schedules of 150 trials per session.

The data from STB and VRB were analyzed both as a function of H_sch_ (the objectively highest value stimulus available) and of H_RL_ (the subjectively highest value stimulus given the animals' choices as derived using a standard Rescola-Wagner learning model with a Boltzmann action selection rule). The reward learning rate (α) was fitted individually to each animal's pre-surgery data using standard nonlinear minimization procedures.

Where appropriate, data from all tasks are reported using parametric repeated-measures ANOVA (see Supplemental Information).

To establish the contribution of choices recently made and rewards recently received on subsequent choices, we performed three separate logistic regression analyses, one for each potential stimulus (A, B, C). This gave us three sets of regression weights, ${\hat{\beta }}_{A},{\hat{\beta }}_{B},{\hat{\beta }}_{C}$ and three sets of covariances, ${\hat{C}}_{A},{\hat{C}}_{B},{\hat{C}}_{C}$. We proceeded to combine the regression weights into a single weight vector using the variance-weighted mean:

$$\hat{\beta }={\left({\hat{C}}_{A}^{-1}+{\hat{C}}_{B}^{-1}+{\hat{C}}_{C}^{-1}\right)}^{-1}\left({\hat{C}}_{A}^{-1}{\hat{\beta }}_{A}+{\hat{C}}_{B}^{-1}{\hat{\beta }}_{B}+{\hat{C}}_{C}^{-1}{\hat{\beta }}_{C}\right)$$

### Surgery

Surgical procedures and histology for these animals have been described in detail elsewhere (Rudebeck et al., 2008). In brief, animals were given aspiration lesions to the OFC using a combination of electrocautery and suction under isoflurane general anesthesia. The lesions were comparable to those reported in Izquierdo et al. (2004), taking the tissue medial to the lateral orbital sulcus up to the gyrus rectus on the medial surface (Figure 1A; see Supplemental Information).

## Supplemental Information

Document S1. Six Figures, Supplemental Results, and Supplemental Experimental Procedures
