# Supplementary material for: Separable Learning Systems in the Macaque Brain and the Role of Orbitofrontal Cortex in Contingent Learning
Source: Neuron. 2010 Mar 25;65(6):927–39. doi: 10.1016/j.neuron.2010.02.027 (PMC3566584; doi:10.1016/j.neuron.2010.02.027)
Supplement: Document S1. Six Figures, Supplemental Results, and Supplemental Experimental Procedures [file mmc1.pdf]

Neuron, Volume *65*

Supplemental Information

Separable Learning Systems in the Macaque Brain and the Role of Orbitofrontal Cortex in Contingent Learning

Mark E. Walton, Timothy E.J. Behrens, Mark J. Buckley, Peter H. Rudebeck, and Matthew F.S. Rushworth

1. Supplemental Data

Supplemental Results

Figure S1 (related to Figure 4).

Figure S2

Figure S3 (related to Figure 5)

Figure S4 (related to Figure 6A)

Figure S5 (related to Figure 6B)

Figure S6

2. Supplemental Experimental Procedures

3. Supplemental References

Supplemental Data

Choice alternation as a function of local reward rate

OFC-lesioned animals demonstrated raised rates of trial-by-trial switching behavior following surgery (Figure 4). However, it is possible that this was simply a consequence of the reversal deficit causing these animals to receive less frequent rewards than they had prior to the reversal. As can be seen in Figure S1A, whereas both groups of animals pre-operatively were increasingly likely to persist with choosing an option as the local reward rate increased, OFC-lesioned monkeys post-operatively did not display this pattern of increased persistence with increasing local reward rate except when the reward rate was at its highest (≥0.7 rewards/choice)(Lesion Group x Surgery x Reward Rate: $F_{8,32}=3.05$, $p=0.011$). This was particularly marked in the post-reversal phase of both conditions.

Importantly, when the data were divided up by whether or not a reward was delivered immediately before a switch, the OFC-lesioned animals displayed a comparable increased propensity to alternate between choices compared to controls following either a positive or negative outcome on the previous trial (Lesion Group x Surgery x Previous Reward and Lesion Group x Surgery x Previous Reward x Reward Rate: both $Fs<2.5$, both $p>0.14$) (Figure S1B). All these effects were replicated if instead rates of switching were investigated as a function of subjective stimulus values rather than local reward rates. This demonstrates that the OFC lesion did not cause a particular problem with monitoring and responding to negative reinforcement or with inhibiting responding to the previously most highly rewarding stimulus (Fellows, 2007; Kringelbach and Rolls, 2004).

Choice alternation as a function of recent reward- and choice-histories

An integrated history of recent rewards is most predictive of the current reward in two situations: when the recent reward rate is very low (as current rewards are very unlikely), and when the recent reward rate is very high (as current rewards are very likely). Data already presented (Figure S1) depicted the monkeys' alternation behavior as a function of local reward rate. At the lowest and highest reward rates, OFC and control patterns of switching are indistinguishable. By contrast, when the local reward rate was at intermediate levels (meaning that the current trial was equally likely to be rewarded or not and could not, therefore, be predicted using the integrated history of reward), the OFC group's switching behavior deviated significantly from that of the control group.

In this vein, we also re-examined whether the OFC-lesioned animals' patterns of response alternation in STB and VRB were being influenced by their recent history of choices by plotting trial-by-trial rates of switching as a function of the number of times prior to a switch that they had selected the same option. In order to obtain sufficient data for this, we collapsed across both phases of the STB and VRB schedules. While an equivalent pattern of significantly increased response alternation was observed in the OFC-lesioned animals following sequences of 1 or 2 choices of the same option (sequence of 1 response type: Lesion Group x Surgery x Value: $F_{4,16}$=7.13, p=0.002; sequence of 2 response types: Lesion Group x Surgery: $F_{1,4}$=10.21, p=0.033), as the sequences increased to 3-5 selections of the same option, the OFC group's likelihood of persisting increased and become indistinguishable from controls (Figure S6). Therefore, as the lesioned animals' choice history became more consistent, their pattern of choices also became more similar to control animals. This again implies that OFC-lesioned animals might be using reinforcement not to update the value of the immediately

preceding chosen option but instead to revalue all the options as a function of recent choice history.

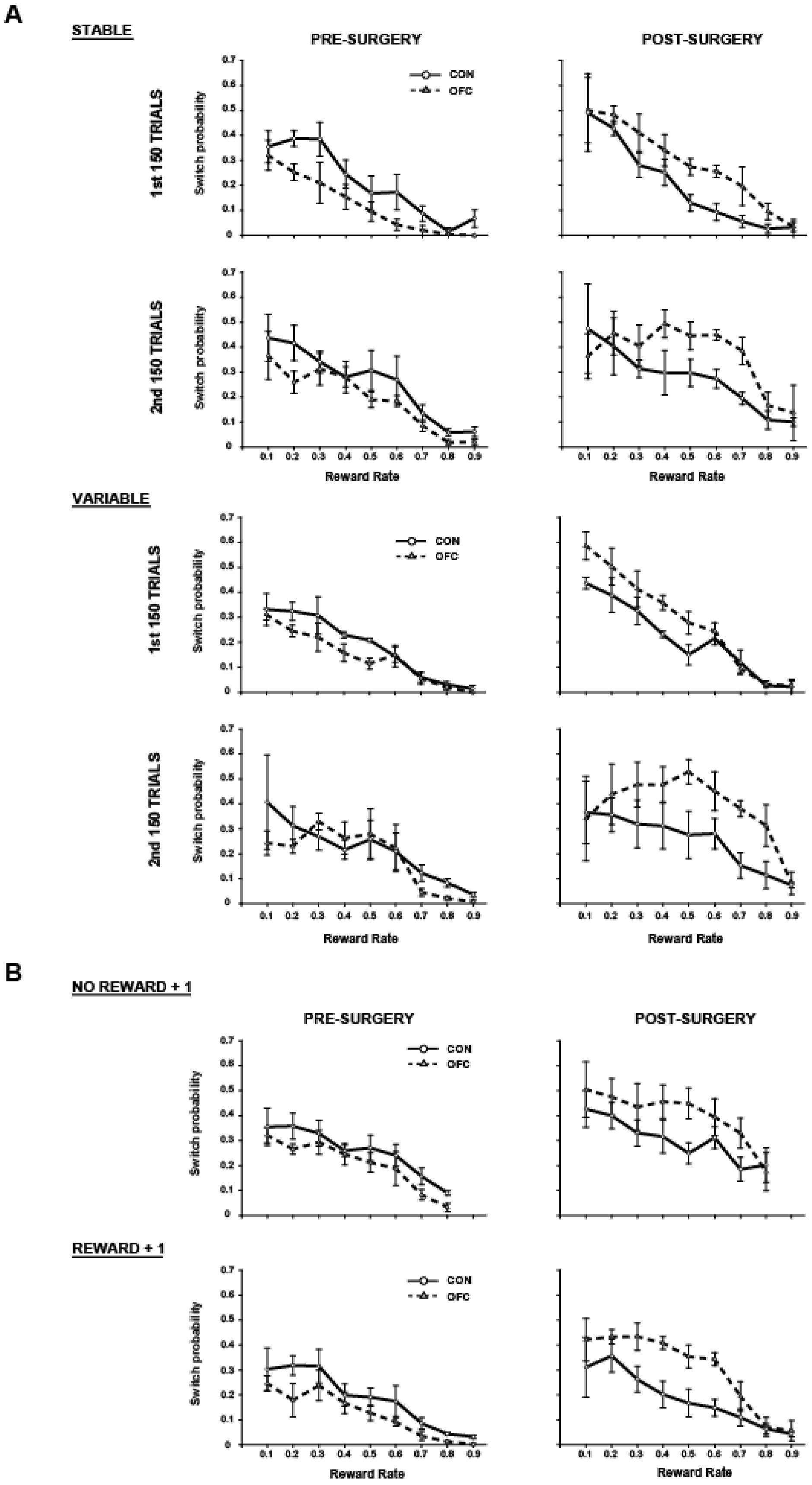
A
STABLE
PRE-SURGERY
POST-SURGERY
1st 150 TRIALS
2nd 150 TRIALS
Switch probability
CON
OFC
Reward Rate
VARIABLE
B
NO REWARD + 1
REWARD + 1

Figure S1 (related to Figure 4). Switching likelihood as a function of recent local reward rate (rewards / trial, averaged over the past 10 trials) divided up (A) by condition (STB, upper panels; VRB, lower panels), by surgery (pre-surgery, left-hand column; post-surgery, right-hand column) and by phase (1st 150 trials, pre-reversal, or 2nd 150 trials, post-reversal) and (B) by whether or not the previous trial was rewarded (no reward on previous trial, upper panels; reward on previous trial, lower panels). Controls = open circles and filled line; OFCs = gray triangle and dashed line.

Reward schedules

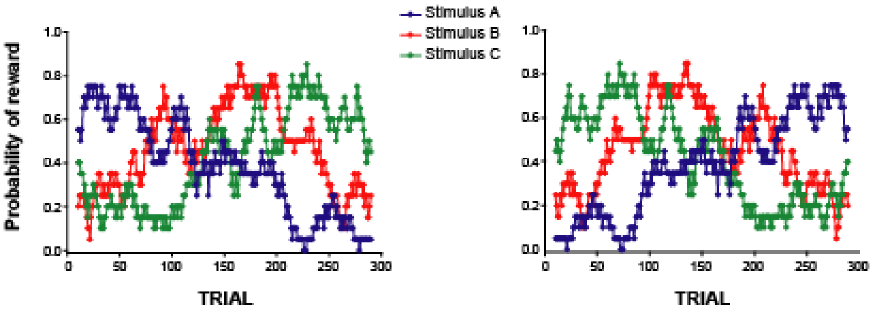

Figure S2. Predetermined reward schedules from two additional 3-armed bandit conditions (which are mirror images of each other, with, for instance, the likelihood of reward on trial 10 in the left-hand condition being identical to trial 290 in the right-hand one). Animals' choices from 5 sessions of testing on both schedules was included in the logistic regression (Figure 5) and reward-/choice-history analyses (Figure 6, S5-6) in order to provide sufficient trials to obtain adequate estimates of the effects of reward- and choice history. Choice behavior in one of the conditions (left-hand panel) has previously been reported in Rudebeck et al. (2008). As before, the schedules determined whether or not reward was delivered for selecting a stimulus (A-C) on a particular trial.

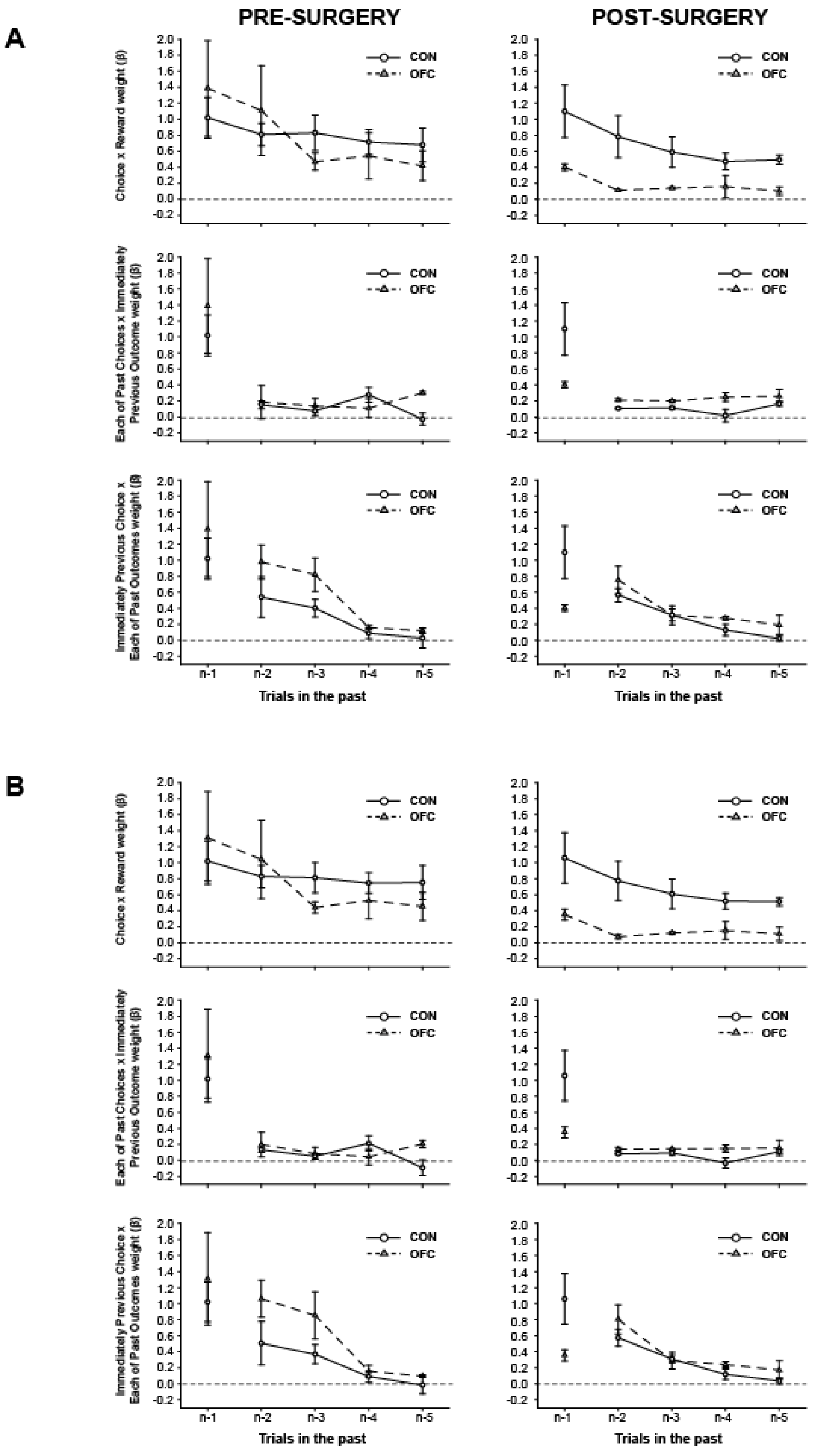

Figure S3 (related to Figure 5). Influence of recent choices and their specific outcomes, each past choice and the previous outcome, and each past outcome and the previous choice on current choice behavior as a function of (A) A choices, and (B) B or C choices only.

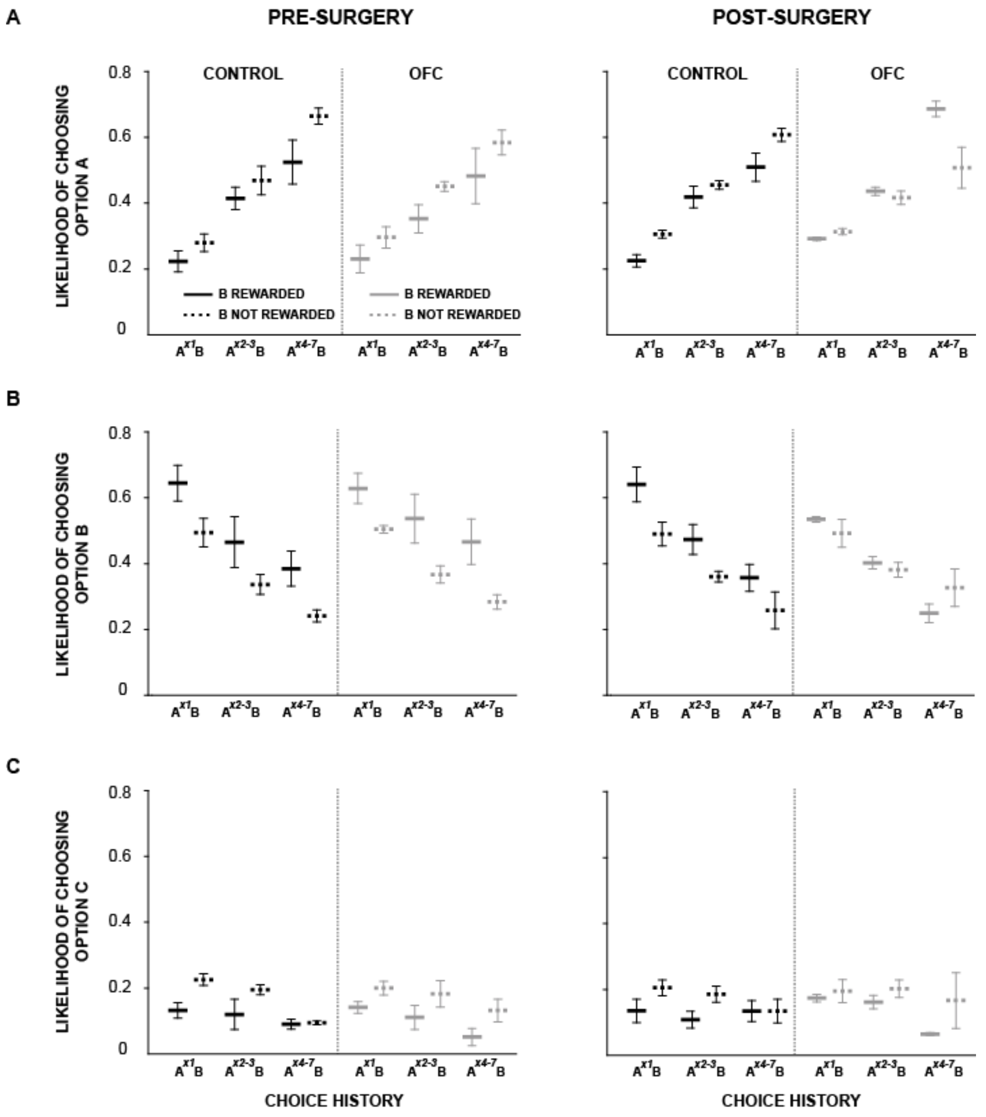

Figure S4 (related to Figure 6A). Influence of past choices of one option (A) on current choice behavior (trial n) in changeable 3-armed bandit tasks as a function of reward received for choosing option B on the previous trial (trial n-1). Note, as elsewhere, *options* A, B, and C do not necessarily refer to selection of *stimuli* A, B, and C but instead to similar patterns of choices (i.e., an "AAB" history can be made up of choices of stimulus AAB, AAC, BBC, BBA, CCB, or CCA). Top row: likelihood of choosing option A on trial n after either receiving a reward (filled line) or not receiving a reward (dashed line) for choosing option B on the previous trial (n-1). Middle row: likelihood of choosing option B on trial n. Bottom row: likelihood of choosing option C on trial n. The data in Figure 5 depicts the above data as the subtraction of (B rewarded - B not rewarded) for each choice history sequence.

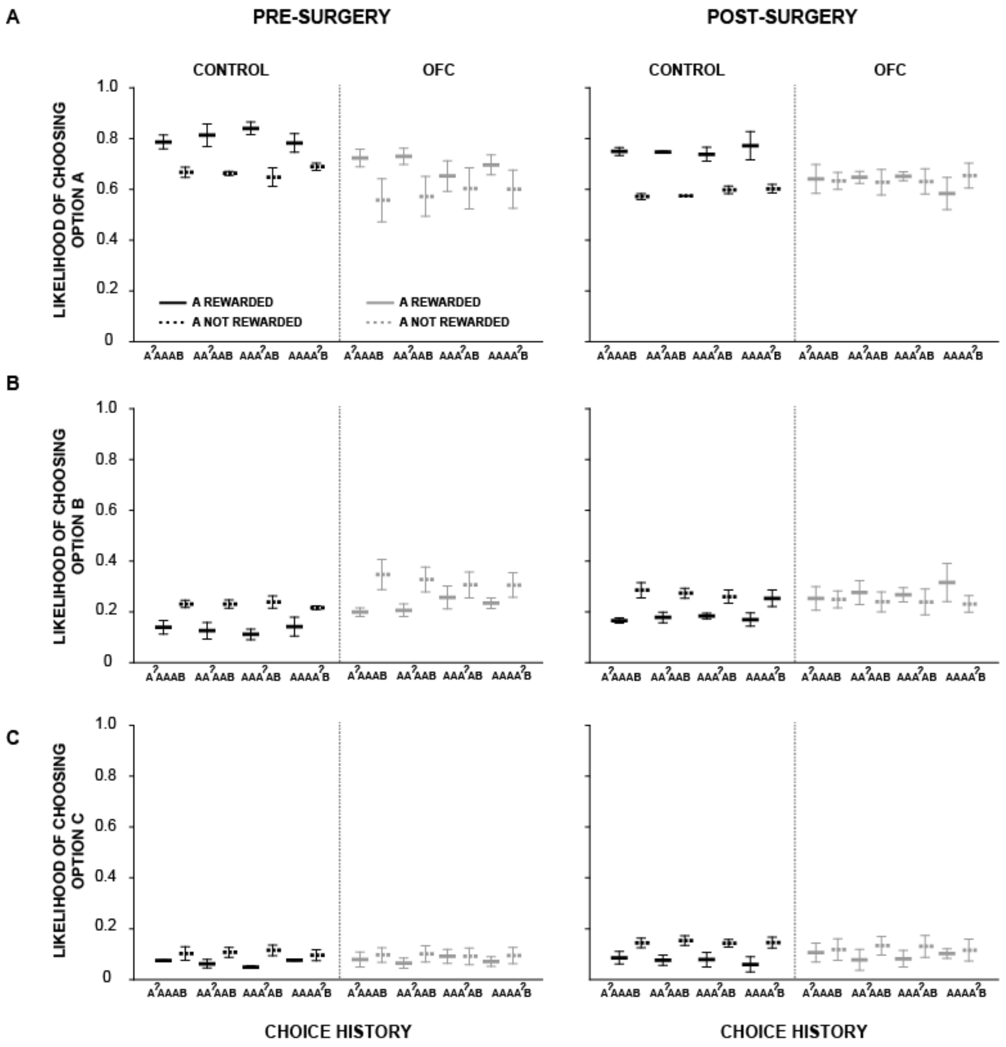

Figure S5 (related to Figure 6B). Likelihood of choosing a particular option on the current trial (n) after having chosen option A on 4 past trials (n-2 to n-5) and then option B on the previous trial (n-1), plotted as a function of reinforcement on one particular A option in the past ($A^?$). Top row = likelihood of choosing option A on trial n when previous A choice ($A^?$) was either rewarded (filled line) or not rewarded (dashed line); middle row = likelihood of choosing option B on trial n; bottom row = likelihood of choosing option C on trial n.

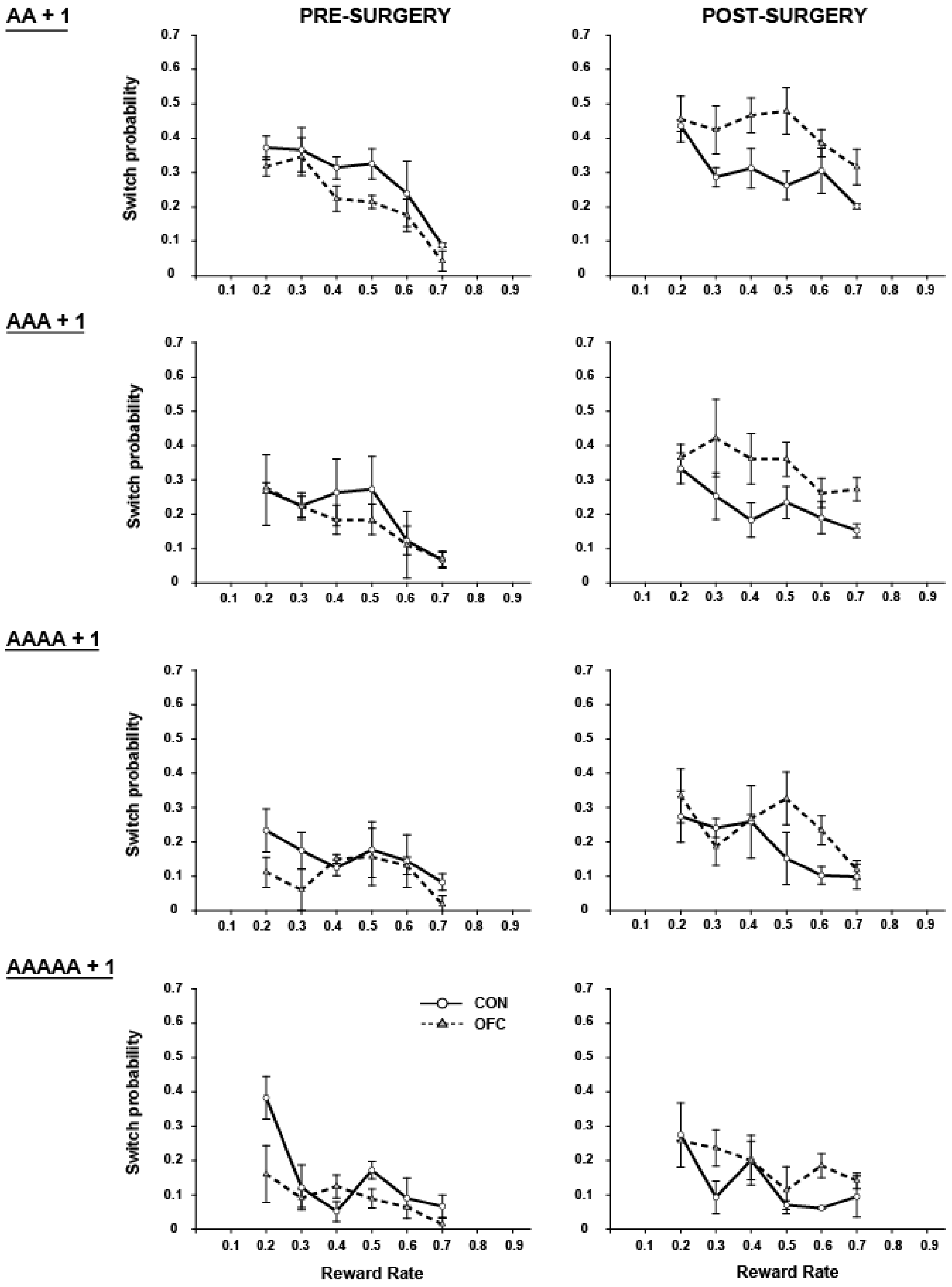

Figure S6. Switching likelihood across all trials of STB and VRB as a function of recent local reward rate (past 10 trials) divided up by uniformity of recent choice history. Top row = likelihood of switching on the trial after having made the same choice twice (AA+1); second row = likelihood of switching having made the same choice three times (AAA+1); third row = likelihood of switching having made the same choice four times (AAAA+1); bottom row = likelihood of switching having made the same choice five times (AAAAA+1). As elsewhere, “A” can refer to selection of stimulus A, B or C with the appropriate choice sequence. Controls = open circles and filled line; OFCs = gray triangle and dashed line.

Supplementary Experimental Procedures

Apparatus

Each monkey sat unrestrained in a wheeled transport cage placed 20cm from a touch-sensitive monitor (38cm wide x 28cm high) in a testing room on which visual stimuli could be presented (8 bit color clipart bitmap images, 128 x 128 pixels) and responses recorded. Rewards (190mg Noyes pellets) were delivered from a dispenser (MED Associates) into a food well immediately to the right of the touch screen. A large metal food box, situated to the left below the touch screen, contained each individual's daily food allowance (given in addition to the reward pellets) consisting of proprietary monkey food, fruit, peanuts and seeds, delivered immediately after testing each day. This was supplemented by a forage mix of seeds and grains given ~6 hours prior to testing in the home cage. Stimulus presentation, experimental contingencies, reward delivery and food box opening was controlled by a computer using in-house software.

Statistical Analyses

Where appropriate, data from STB and VRB are reported using parametric repeated-measures ANOVA, with within-subjects factors of Surgery (2 levels: Pre- or Post-Surgery), Condition (2 levels: STB or VRB), and Testing Session (5 levels: Session 1–5). Analyses of performance before and after reversal in identity of H_{sch} included the factor of Phase (2 levels: 1st or 2nd 150 trials in a session), and response alteration analyses included local reward rate – the average likelihood of reward per trial across the previous 10 trials – or subjective reward value (both 9 levels: 0.1–0.9). FIXED schedules were analyzed comparably, though without the factor of Surgery (as all testing occurred post-surgery). Performance criterion measures used geometric means of the number of trials

taken to choose the H_{sch} option on ≥65% trials over the 5 sessions to account for skew induced by days on which no criterion was reached (and so a maximum of 140 trials was logged). These were then compared with separate Mann-Whitney tests as to account for violations in normality in the data.

Logistic Regression

In order to ascertain the influence of specific choice-outcome associative learning and associations based on recent choice- and reward-histories, we performed three separate logistic regression analyses, one for each potential stimulus (A,B,C). This gave us three sets of regression weights ,$\hat{\beta}_A, \hat{\beta}_B, \hat{\beta}_C$ and three sets of covariances $\hat{C}_A, \hat{C}_B, \hat{C}_C$. The regression weights into a single weight vector using a variance-weighted mean (Lindgren, 1993):

$$\hat{\beta} = \left(\hat{C}_A^{-1} + \hat{C}_B^{-1} + \hat{C}_C^{-1}\right)^{-1}\left(\hat{C}_A^{-1}\hat{\beta}_A + \hat{C}_B^{-1}\hat{\beta}_B + \hat{C}_C^{-1}\hat{\beta}_C\right)$$

However, results were essentially identical if we instead used the arithmetic mean:

$$\hat{\beta} = \frac{\left(\hat{\beta}_A + \hat{\beta}_B + \hat{\beta}_C\right)}{3}.$$

The remainder of this section will describe the analysis of only the "A" choices, and imply corollaries for B and C.

We used as the dependent variable a binary indicator variable which took the value 1 whenever the animal chose A and the value 0 whenever the animal did not choose A (i.e. when they chose B or C). We then formed independent variables (IVs) as based on all

possible combinations of recent past choices and recent past rewards (trials n-1, n-2,…,n-6)(Figure 5A). Each IV took the value 1 when, for the particular choice-outcome interaction, the animal chose A and was rewarded, the value -1 when the animal chose B or C and was rewarded, and the value 0 when there was no reward (Figure 5B). We then fit a standard logistic regression with these 36 IVs to give us estimates of $\hat{\beta}_A$ and $\hat{C}_A$.

The data depicted in Figure 5 are the influence on trials n-1 to n-5 when A was rewarded and Bs or Cs were unrewarded. However, the data were essentially unaffected when only A rewards or B,C rewards were included in the design matrix (Figure S5). As the 5th row and column is the only one in the matrix that contains variance from the choices and outcomes on trial n-5, it will therefore be sensitive to any longer-term choice/reward trends. To avoid this effect, we therefore included a 6th row/column in the matrix describing choices and outcomes n-6. These regressors were included as confound regressors for the 5th row and are therefore not shown.

Surgery and Histology

Surgical procedures in these animals have been described in detail elsewhere (Rudebeck et al., 2008). The lesions were intended to be comparable to those reported in Izquierdo et al. (2004), taking the tissue medial to the lateral orbital sulcus up to the gyrus rectus on the medial surface. The rostral and caudal boundaries were by imaginary perpendicular lines connecting, respectively, the rostral- and caudal-most points of the medial and lateral orbital sulci. Immediately following surgery and for ~5 days subsequently, animals were given non-steroidal anti-inflammatory analgesic (0.2 mg/kg meloxicam, orally) and antibiotic (8.75 mg/kg amoxicillin, orally), and were allowed at

least 3 weeks for full recovery prior to post-operative testing. Post-operative data collection for the experiments reported here started between 8-12 weeks after surgery.

Following completion of all testing, animals were deeply anesthetized with sodium pentobarbitone and perfused with 90% saline and 10% formalin, their brains removed and placed in 10% sucrose formalin until they sank. The brains were subsequently blocked in the coronal plane at the level of the most medial part of the central sulcus. Each brain was cut in 50 μm coronal sections, with every 10th section retained and stained with cresyl violet for analysis of the extent of the lesion.

The extent of the OFC lesions has also been described in detail elsewhere (Rudebeck et al., 2008). In brief, the lesions were largely as intended, reliably destroying the tissue in Walker's areas 11 and 13 in all cases (Walker, 1940) (Figure 1A). On the lateral extent, area 12 was largely spared except for part of this region in the left hemisphere of one animal. The lesion was more variable in the extent to which area 14 on the medial surface was damaged, with anterior medial sections largely spared along with posterior parts of the gyrus rectus.

Supplemental References

Lindgren, B.W. (1993). Statistical Theory, 4th Edition edn (New York: Chapman & Hall).

Walker, A.E. (1940). A cytoarchitectural study of the prefrontal area of the macaque monkey. J Comp Neurol *73*, 59-86.
